# Clinical Audit of Dengue Related Deaths in 2011-Mayo Hospital Lahore Pakistan

**DOI:** 10.12669/pjms.335.13051

**Published:** 2017

**Authors:** Somia Iqtadar, Nabeel Akbar, Mehreen Mehmood, Sajid Abaidullah

**Affiliations:** 1 Somia Iqtadar, FCPS. Department of Medicine, Mayo Hospital, Lahore, Pakistan; 2 Nabeel Akbar, MBBS. Department of Cardiology, Punjab Institute of Cardiology, Lahore, Pakistan; 3 Mehreen Mehmood, MBBS. Department of Medicine, Mayo Hospital, Lahore, Pakistan; 4 Sajid Abaidullah, FCPS. Department of Medicine, Mayo Hospital, Lahore, Pakistan

**Keywords:** Dengue, Complications, Co-morbidities, Mortality, Fluid overload, Massive bleeding

## Abstract

**Background and Objective:**

Dengue infection has evolved into an epidemic during last few years in Pakistan and has been associated with poor outcomes. Literature with respect to mortality risk factors in Dengue infection is not sufficient. This compelled us to conduct this study to find out major contributory factors to death in patients with dengue viral infection at one of Asia’s ancient hospital setting with an aim to recognize complications at earliest and improve case management in future.

**Methods:**

A retrospective observational study of 95 adult dengue deaths was performed at Mayo Hospital Lahore from July 1st 2011 to 31st December 2011 during a major dengue epidemic. Patients who tested positive by dengue IgM in the presence of acute fever fulfilling the World Health Organization criteria for Dengue Fever, Dengue Hemorrhagic Fever or Dengue Shock Syndrome and died within same setting, were included. Data regarding demographic profile, clinical and laboratory parameters along with treatment details were obtained and analysed. All records examined were anonymized.

**Results:**

Median age was 36 years (range13-80 years) among 95 deaths due to Dengue. Male gender comprised 60 (63.1%). Co-morbidities existed in 74 (77.9%) with hypertension in 21 (22.1%) diabetes in 11 (11.58%), liver disease in 9 (9.47%) and ischemic heart disease in 8(8.4%) cases. Patients presented at second day of fever for admission (range 1-8 days) and death occurred at a median of 4 days (range 30 minutes to 23 days). Hospital stay was for less than a week for seventy nine (83.2%) patients and 16 (16.8%) were admitted for more than 7 days. Critical care was required in 67(71%). Severe hepatitis occurred in 41 (43.1%), acute renal impairment occurred in 32 (33.7%) and disseminated intravascular coagulation in 16 (16.8%). Deaths were due to prolonged shock 49 (51.5%) fluid overload 46 (48.4%) and massive bleeding 18(19%) leading to organ failure.

**Conclusion:**

Decompensated shock complicated by either massive plasma leakage, frank bleeding, multi organ failure or deranged clotting profile results in enhanced mortality in Dengue infection. Co-morbidities especially Diabetes are poor prognostic factors in predicting Dengue mortality.

## INTRODUCTION

Approximately 3.6 billion people residing in the tropics and subtropics are affected by Dengue Infection.^1^ This rapidly increasing arthropod-borne disease has four serotypes (DENV-1, DENV-2, DENV-3 and DENV-4). The spectrum of illness ranges from a mild self-limiting illness to dengue fever (DF), in severe cases, dengue hemorrhagic fever (DHF) and dengue shock syndrome (DSS).^2^ Annually 24000 deaths have been attributed to Dengue with continuous surge in numbers each successive year.^3^

Dengue infection epidemics are on rise in the South East Asian belt. India, Pakistan and Sri Lanka have greater chunk of people suffering from this infection.^3^ A recent study done in a tertiary care setting showed that there is a rise in number of DHF and DSS cases in Pakistan.^4^

Primary prevention of dengue through vector control activities has not been able to achieve desired results yet.^5^ Currently there is no anti-viral treatment to treat Dengue. However, recently a vaccine has been launched to prevent dengue with variable protection rate against different subtypes.

Improvisation in clinical management in last few years has been able to effectively reduce mortality from figures as high as 20% to less than 1%.^6^ Several factors have been affiliated to Dengue infection as poor prognostic markers. Various studies done in Africa^5^, Australia, America, South Asia.^7^,^8^ have documented risk factors that lead to poor prognosis. Elderly people are more prone to have severe form of illness as reported in study done in Taiwan.^9^ Another study conducted in Cuba documented that DF and DHF has increase mortality rates in extremes of ages.^10^ A study done in Singapore showed that dengue infection in presence of chronic illness such as chronic renal failure or chronic pulmonary obstructive disease were likely to have poor outcome.^11^ Secondary bacteremia was also associated with high mortality rates as per study done in Taiwan^9^. Upper GI bleed^12^, thrombocytopenia and leukocytosis.^13^ and tachycardia on admission.^14^ are among the important mortality indicators.

Recently dengue infection has unfolded into an epidemic in Pakistan as reported in different studies. Our physicians faced a great challenge to tackle this large pool of patients in 2011 and led to high case fatality rate. We therefore conducted this study to find out major contributory factors to death in patients with dengue infection at a tertiary care center with an aim to improve case management in future.

## METHODS

We performed retrospective observational study of 95 adult dengue deaths at Mayo hospital Lahore from 1st July 2011 to 31st December 2011 during a major dengue epidemic. Mortality records were scrutinized and cases with positive dengue serology were identified. Patients who tested positive by dengue IgM (ELISA) in the presence of acute fever fulfilling the WHO SEARO criteria for DF DHF or DSS, and died within the same hospital admission, were included.

Patients’ demographic profile, epidemiological, serial clinical, laboratory and radiological record was obtained. Existing co-morbidities and treatment details along with disease outcome of the cases were recorded. The duration of acute illness was calculated by noting date of onset of fever to date of discharge from hospital. Ethical approval was procured for the study from the Institutional Review Board (IRB) of the hospital. All records examined were anonymized. According to WHO guidelines DF clinically required the presence of fever and two or more of clinical signs or symptoms including headache, retro-orbital pain, myalgia, arthralgia, rash, hemorrhagic manifestations and leukopenia; DHF was labelled if DF patients had thrombocytopenia ≤100 ×10^9/L, any bleeding, and plasma leakage manifesting as either hematocrit change of ≥20%, fluid accumulation detected clinically or radiologically in the form of pleural effusion or ascites. DSS required signs of circulatory failure in patients with DHF. These include rapid and weak pulse with narrow pulse pressure <20 mmHg, or hypotension for age. Severe hepatitis was labelled when serum alanine or aspartate transaminase were ≥300 units/L.

Acute renal impairment was defined as serum creatinine > 2 times upper limit of normal. Shock was labelled when there was tachycardia, weak or undetectable pulse, narrow pulse pressure ≤20 mmHg, or systolic blood pressure <90 mmHg or unrecordable blood pressure. Markers of shock also included cold and clammy extremities and capillary refill time > 2 seconds. Fluid overload was labelled when there was free fluid in the body confirmed on clinical examination or radiological evidence. Severe bleeding was defined blood loss manifesting as hematemesis, melena, menorrhagia and bleeding per rectum requiring blood transfusion of packed red blood cells. Disseminated intravascular coagulation (DIC) was diagnosed where there was prolongation of prothrombin time (PT) and activated partial thromboplastin time (APTT) in addition to thrombocytopenia in a dengue patient.

## RESULTS

Ninety five adult dengue deaths were reported, of which median age turned out to be 36 years (range 13-80 years). Sixty were male (63.1%). Co-morbidities existed in 74 (77.9%) with hypertension, diabetes, liver disease and ischemic heart disease (IHD) topping the order. Table-I Other minor illnesses were reported in 25(26.3%) patients. Patients presented for admission at a median of two days (range 1-8 days) from first day of appearance of fever and death occurred at a median of four days (range 30 minutes to 23 days). Seventy nine (83.2%) patients were hospitalized for less than a week and 16 (16.8%) were admitted for more than seven days. Intensive care admission was required in 67(71%). Severe hepatitis occurred in 41 (43.1%), acute renal impairment occurred in 32 (33.7%) and DIC in 16 (16.8%). Two patients were offered dialysis support while mechanical ventilation was done in 23 (24.2) and inotropic support was given in 24 (25.3%). Blood and blood components were transfused in 80 (84.2%) patients out of which 45 (47.4%) received whole blood. Platelet transfusions were given in 21 (22.1%) patients while 7 (7.5%) patients received packed red cells. FFP were given in 7 (7.3%) patients. Deaths were due to prolonged shock 49 (51.5%) fluid overload 46 (48.4%) and massive bleeding 18 (19%) leading to organ failure. Dengue was stated as primary cause of death in 21 subjects and 74 deaths were labelled as dengue with co-morbidities. Autopsy was not performed in any of these cases.

**Table-I:** Co-morbidities along with dengue fever.

*No.*	*Comorbidities*	*Cases (Percentages)*
1	Hypertension	21(22.1)
2	Diabetes	11(11.58)
3	Liver Disease	9(9.47)
4	Ischemic heart disease	8(8.4)

## DISCUSSION

Dengue was first time reported in Pakistan in 1994.^15^ Since then dengue virus has become endemic in Pakistan with small yearly outbreaks in different regions of the country. As per provincial health department data 12,682 patients were admitted in hospitals all over Punjab during a period of six months July-Dec 2011. Out of these there were 350 reported deaths in the whole Province. Major patient load was borne by Mayo Hospital Lahore, largest tertiary care hospital of the Province with 6,115 admitted cases and 95 reported deaths.

Case review of Mayo Hospital record showed that there were majority of adult male patients. This male preponderance, especially in Asian countries, is in agreement with other studies conducted in Philippines and Singapore.^16^-^18^ It may be explained due to more outdoor exposure among adult males. Contrasting results are seen in studies conducted in South America where either female proportion is predominant or equal to males.^19^ Since the data collected and interpreted is taken from hospital setting so it is liable to certain limitations threaded to surveillance data such as underreporting, misreporting and reporting biases. Due to existence of subclinical spectrum of Dengue Infection most of the cases do not present to Health care facilities hence, reporting bias results. Similarly, if adult males show up in hospitals seeking medical care more than females then false male preponderance may result even in the absence of gender difference in reality.^20^

In our study, 77.9% patients had co-morbidities. This observation is similar to other studies reporting large number of co morbidities in dengue deaths in America and Africa.^21^ In our data Diabetes mellitus remained the most important comorbid disease. Sudden disequilibrium in comorbid diseases play equal or more part in deaths related to Dengue.

Most of the admissions were done at day two of illness. Some of the patients who presented in critical phase of disease deteriorated rapidly and four patients died within 24 hours of admission. Our observation is in line with an earlier study published from Singapore. In this study seven deaths were reported with mean period of illness prior to hospitalization of 4.8 days.^14^ Presenting to hospital late in course of illness increases the likelihood of mortality in Dengue Infection.^22^

Most common cause of death reported in our study was prolonged shock. Hypotension secondary to shock, altered sensorium and seizures have previously been reported as poor prognostic factors in dengue infection.^4^ Prolonged duration of shock or normal to low hematocrit in DHF/DSS during critical phase heralds underlying bleed. Duration of hypoperfusion of tissues is the deciding factor for multi-organ failure especially in cases of decompensated shock.

Second most common cause of death reported in our study was fluid overload. This was related to overuse of IV fluids including blood and blood products with contribution of renal and liver dysfunction. Studies show that respiratory deterioration mainly due to fluid overload plays important part in increasing death rate in DHF.^23^

The third cause identified in mortality review was severe bleeding. Spontaneous bleeding mostly due to low platelet count is a common complication in dengue patients and is associated with high mortality.^24^ Aspirin which is a commonly used antiplatelet drug in IHD patients, may lead to increased probability of bleeding. Use of non-steroidal anti-inflammatory agents that are notorious for causing platelet dysfunction may be implicated in the pathogenesis of bleeding in DHF/DSS. Platelet dysfunction also occurs in renal disease and liver impairment and effects coagulation leading to more chances of fatal bleeding in dengue patients. Bleed via rectum, vagina and upper gastrointestinal tract were major sources of bleeding in our study.

### Limitations of the Study

One of the limitations of our retrospective descriptive study is dependence on clinician’s attention to details and thorough documentation especially for patients who presented in critical phase and died within 24 hours. Data regarding dengue serotype and primary versus secondary dengue infection was lacking. In addition, this is a review of handful of fatal cases reported from a single tertiary care hospital, which may introduce bias in sample selection. This issue can be addressed by doing multi center case control study comparing both fatal and non-fatal cases and evaluating differences in clinical and laboratory findings of the two groups.

## CONCLUSION

Plasma leakage, frank bleeding, multi organ failure or deranged clotting profile complicates decompensated shock consequently resulting in enhanced mortality in Dengue infection. Among the poor prognostic factors that predicts Dengue mortality, co-morbidities especially Diabetes play important role.

### Authors’ Contribution

**SI** conceived, designed, experimented patient recruitment and wrote manuscript.

**NA** did experimentation, analysis and interpretation of data.

**MM** did editing and manuscript writing.

**SA** did patient recruitment and initial review.

## References

[1] Gubler DJ (2012). The economic burden of dengue. Am J Trop Med Hyg.

[2] World Health Organization (1997). Dengue hemorrhagic fever:diagnosis, treatment, prevention and control.

[3] Barboza P, Tarantola A, Lassel L, Mollet T, Quatresous I, Paquet C (2008). Emerging viral infections in South East Asia and the Pacific region. Med Mal Infect.

[4] Wasay M, Channa R, Jumani M, Zafar A (2008). Changing patterns and outcome of Dengue infection;report from a tertiary care hospital in Pakistan. J Pak Med Assoc.

[5] Adam I, Jumaa AM, Elbashir HM, Karsany MS (2010). Maternal and perinatal outcomes of dengue in Port Sudan, Eastern Sudan. Virol J.

[6] Barnes WJ, Rosen L (1974). Fatal hemorrhagic disease and shock associated with primary dengue infection on a Pacific island. Am J Trop Med Hyg.

[7] Raheel U, Faheem M, Riaz MN, Kanwal N, Javed F, Qadri I (2010). Dengue fever in the Indian subcontinent:an overview. J Infect Dev Ctries.

[8] Anders KL, Nguyet NM, Chau NV, Hung NT, Thuy TT, Lien le B (2011). Epidemiological factors associated with dengue shock syndrome and mortality in hospitalized dengue patients in Ho Chi Minh City, Vietnam. Am J Trop Med Hyg.

[9] Lee K, Liu JW, Yang KD (2008). Clinical and laboratory characteristics and risk factors for fatality in elderly patients with dengue hemorrhagic fever. Am J Trop Med Hyg.

[10] Guzmán MG, Kouri G, Bravo J, Valdes L, Susana V, Halstead SB (2002). Effect of age on outcome of secondary dengue 2 infections. Int J Infect Dis.

[11] Lahiri M, Fisher D, Tambyah PA (2008). Dengue mortality:reassessing the risks in transition countries. Trans R Soc Trop Med Hyg.

[12] Chua MN, Molanida R, De Guzman M, Laberiza F (1992). Prothrombin time and partial thromboplastin time as a predictor of bleeding in patients with dengue hemorrhagic fever. Southeast Asian J Trop Med Public Health.

[13] Lee K, Liu JW, Yang KD (2012). Fatal dengue hemorrhagic fever in adults:emphasizing the evolutionary pre-fatal clinical and laboratory manifestations. PLoS Negl Trop Dis.

[14] Ong A, Sandar M, Chen MI, Sin LY (2007). Fatal dengue hemorrhagic fever in adults during a dengue epidemic in Singapore. Int J Infect Dis.

[15] Gubler DJ, Clark GG (1995). Dengue/dengue hemorrhagic fever:the emergence of a global health problem. Emerg Infect Dis.

[16] Anker M, Arima Y (2011). Male-female differences in the number of reported incident dengue fever cases in six Asian countries. Western Pac Surveill Response J.

[17] Yew YW, Ye T, Ang LW, Ng LC, Yap G, James L (2009). Seroepidemiology of dengue virus infection among adults in Singapore. Ann Acad Med Singapore.

[18] Communicable Diseases Surveillance in Singapore 2009 | Ministry of Health [Internet] (2010). Moh.gov.sg.

[19] Günther J, Ramírez-Palacio LR, Pérez-Ishiwara DG, Salas-Benito JS (2009). Distribution of dengue cases in the state of Oaxaca, Mexico, during the period 2004–2006. J Clin Virol.

[20] Endy TP, Chunsuttiwat S, Nisalak A, Libraty DH, Green S, Rothman AL, Vaughn DW, Ennis FA (2002). Epidemiology of inapparent and symptomatic acute dengue virus infection:a prospective study of primary school children in Kamphaeng Phet, Thailand. Am J Epidemiol.

[21] Rigau-Pérez JG, Laufer MK (2006). Dengue-related deaths in Puerto Rico 1992–1996: diagnosis and clinical alarm signals. Clin Infect Dis.

[22] Montenegro D, Lacerda HR, Lira TM, Oliveira DS, Lima AA, Guimarães MJ (2006). Clinical and epidemiological aspects of the dengue epidemic in Recife, PE 2002. Revista da Sociedade Brasileira de Medicina Tropical.

[23] Sam SS, Omar SF, Teoh BT, Abd-Jamil J, AbuBakar S (2013). Review of dengue hemorrhagic fever fatal cases seen among adults:a retrospective study. PLoS Negl Trop Dis.

[24] Díaz-Quijano FA (2008). Predictors of spontaneous bleeding in dengue patients:a systematic review of the literature. Invest Clin.

